# The Impact of Bone Marrow Involvement on Prognosis in Diffuse Large B-Cell Lymphoma: An 18F-FDG PET/CT Volumetric Segmentation Study

**DOI:** 10.3390/cancers16223762

**Published:** 2024-11-07

**Authors:** Andrej Doma, Andrej Studen, Barbara Jezeršek Novaković

**Affiliations:** 1 Department of Nuclear Medicine, Institute of Oncology Ljubljana, 1000 Ljubljana, Slovenia; 2 Faculty of Medicine, University of Ljubljana, 1000 Ljubljana, Slovenia; 3 Experimental Particle Physics Department, Jožef Stefan Institute, 1000 Ljubljana, Slovenia; 4 Faculty of Mathematics and Physics, University of Ljubljana, 1000 Ljubljana, Slovenia; 5 Division of Medical Oncology, Institute of Oncology Ljubljana, 1000 Ljubljana, Slovenia

**Keywords:** 18F-FDG, PET/CT, diffuse large B-cell lymphoma, bone marrow, MTV, survival

## Abstract

Background: This study assessed the prognostic value of tumor burden in bone marrow (BM) and total disease (TD), as depicted on 18F-FDG PET/CT in 140 DLBCL patients, for complete remission after first-line systemic treatment (iCR) and 3- and 5-year overall survival (OS3 and OS5). Methods: Baseline 18F-FDG PET/CT scans of 140 DLBCL patients were segmented to quantify metabolic tumor volume (MTV), total lesion glycolysis (TLG), and SUVmax in BMI, findings elsewhere (XL), and TD. Results: Bone marrow involvement (BMI) presented in 35 (25%) patients. Median follow-up time was 47 months; 79 patients (56%) achieved iCR. iCR was significantly associated with TD MTV, XL MTV, BM PET positivity, and International Prognostic Index (IPI). OS3 was significantly worse with TD MTV, XL MTV, IPI, and age. OS5 was significantly associated with IPI, but not with MTVs and TLGs. Univariate factors predicting OS3 were XL MTV (hazard ratio [HR] = 1.29), BMI SUVmax (HR = 0.56), and IPI (HR = 1.92). By multivariate analysis, higher IPI (HR = 2.26) and BMI SUVmax (HR = 0.91) were significant independent predictors for OS3. BMI SUVmax resulted in a negative coefficient and hence indicated a protective effect. Conclusions: Baseline 18F-FDG PET/CT MTV is significantly associated with survival. BMI identified on 18F-FDG PET/CT allows appropriate treatment that may improve survival.

## Simple Summary

Bone marrow (BM) involvement in diffuse large B-cell lymphoma (DLBCL) impacts disease staging, treatment, and prognosis. This study examined the prognostic value of several volumetric FDG PET/CT biomarkers in DLBCL. It compared whole-body and BM tumor burdens with patient outcomes, including remission and three-year and five-year survival rates. The study also evaluated the International Prognostic Index and its components to develop a predictive model combining FDG PET/CT and traditional biomarkers. In a group of 140 DLCBL patients, we confirmed that tumor burden on FDG PET/CT was an independent disease outcome predictor significantly associated with survival, confirming its potential to improve disease staging, prognosis accuracy, and treatment. Surprisingly, BM infiltration on baseline FDG PET/CT has been associated with improved survival outcomes. We demonstrated that having a positive BM in PET, although initially indicating a worse outcome, allows for identifying patients who benefit from more intensive therapy that may improve survival.

## 1. Introduction

Diffuse large B-cell lymphoma (DLBCL) is the most common subtype of aggressive non-Hodgkin lymphoma in adults in the western world [1], with annual incidence rates of 6.87 and 6.33 per 100,000 person-years in the United States [2] and Slovenia [3], respectively. Initial treatment with R-CHOP (rituximab, cyclophosphamide, doxorubicin, vincristine, and prednisone) results in complete remission (CR) in over 75% of patients with advanced-stage disease [4,5]. Patients who remain event-free 24 months after treatment, have mortality rates similar to the general population within a few years [6]. However, 10–15% of patients fail initial therapy, and 20–25% relapse, with salvage therapies often showing limited success [7,8].

Accurate prognostic markers are crucial for identifying patients at high risk of progression or relapse, allowing for more intensive or novel therapeutic approaches. Bone marrow involvement (BMI) in DLBCL significantly impacts disease staging, treatment, and prognosis [9]. Studies indicate that patients with BMI have poorer progression-free survival (PFS) and overall survival (OS) [10]. BMI is also indirectly incorporated into the International Prognostic Index (IPI) [11].

18F-Fluorodeoxyglucose Positron Emission Tomography-Computed Tomography (FDG PET/CT) is routinely used to measure parameters from all detectable lymphoma lesions in the body. High baseline metabolic tumor burden, measured by metabolic tumor volume (MTV) and total lesion glycolysis (TLG) on FDG PET/CT, are associated with worse prognosis in DLBCL [12,13,14,15,16,17]. While some studies have assessed the prognostic value of separate volumetric FDG PET/CT parameters for specific organs involved in DLBCL (e.g., colon, extranodal, nodal, and spleen) [18,19], no study has successfully assessed the prognostic impact of BMI tumor burden utilizing the manual segmentation of separate quantitative BMI volumetric parameters MTV and TLG on FDG PET/CT. While the accurate segmentation of pathological FDG PET/CT uptake is time-consuming and varies among physicians, automated tools often fail to distinguish between tumor and physiological uptake and therefore the full automation of BMI segmentation [20] might be unreliable [21]. Consequently, the prognostic impact of separate volumetric FDG PET/CT parameters of BMI remains unexplored.

Our study aimed to analyze the relationship between baseline FDG PET/CT volumetric biomarkers of total disease (TD) in whole body and separate bone marrow (BM) tumor burdens and patient outcomes, including complete remission after first-line systemic treatment (iCR), overall survival at 3 years (OS3), and overall survival at 5 years (OS5). We compared the prognostic value of baseline FDG PET/CT volumetric biomarkers with established factors like IPI and sought to develop the best model for predicting survival by combining FDG PET/CT and traditional biomarkers. To better understand the prognostic value of the IPI score, we analyzed its individual constituents, including stage, World Health Organization performance status (WHO), and age, along with MIB-1 immunohistochemical proliferation index (MIB-1).

## 2. Materials and Methods

### 2.1. Patients

This retrospective study included 507 patients referred to the Institute of Oncology Ljubljana with a suspected diagnosis of DLBCL from January 2016 to December 2020. Inclusion criteria were histologically confirmed DLBCL, stage II–IV disease, pre-treatment FDG PET/CT, and baseline BM biopsy (BMB). Exclusion criteria included age < 18 or >80, stage I disease, central nervous system (CNS) involvement, and concurrent or prior malignancies, including low-grade lymphoma. Treatment involved 6 or 8 cycles of R-CHOP or similar therapy according to local guidelines, followed by radiotherapy to residual disease, if needed [22,23]. Patient outcomes were assessed using OS and iCR rates. OS was defined as the time from diagnosis to death from any cause. Specific mortality causes to assess the prognostic impact of different biomarkers were not considered, but instead the all-cause mortality was used, where no distinction was made between deaths directly attributed to DLBCL and those from unrelated causes. The 3-year and 5-year OS rates were calculated. iCR prior to radiotherapy was defined according to Lugano criteria [23].

### 2.2. Imaging Protocol

PET/CT scans were performed using a Siemens Biograph mCT40 PET/CT, following current guidelines [24]. Patients fasted for 6 h before the examination, with blood sugar levels below 7 mmol/L before ^18^F-FDG injection; intravenous insulin was administered if necessary. ^18^F-FDG activity of 3.7 MBq/kg was administered intravenously 1 h prior to imaging. A tip-of-the-head to mid-thigh PET/CT scan with arms raised was performed, using the following settings: system-regulated current and voltage based on a reference kV value of 100 kV and a reference mAs value of 80 mAs; beam width: 16 × 1.2 mm; pitch: 1.2; PET acquisition time: 2 min/bed position.

An experienced nuclear medicine physician with 15 years of oncological PET/CT experience interpreted and segmented FDG PET/CT images. Pretreatment FDG PET/CT Digital Imaging and Communications in Medicine (DICOM) images were exported to the 3D Slicer suite image processing platform (version 5.0.2, http://www.slicer.org accessed on 24 June 2022). The Slicer3D software suite is a quantitative image analysis tool with well-documented use as a segmentation tool in oncology [25,26,27]. In this study, slicer3D was used with pyRadiomics feature extractor to determine quantitative measures (MTV, TLG, and SUVmax) using shape and first order features (volume, energy, and maximum, respectively) [27,28]. The manual volume of interest (VOI) delineation of two sets of segments in (1) all pathological BMI and (2) infiltrates elsewhere in the body excluding BMI (XL), were performed using a fixed threshold of SUV > 4.0 g/mL, with no minimal volume threshold, as recommended by several authors as the optimal segmentation method for assessing baseline disease burden prognostication in DLBCL patients [29,30]. TD segments were determined as a sum of XL and BMI VOIs. Care was taken not to include areas of physiological activity or benign processes. Only increased uptake in the medullary cavity of the bone was included in the BMI VOI, using co-registered CT images for anatomical guidance. Bone or BM involvement by spread from a contiguous non-skeletal site was excluded from BMI. Additionally, 1 cm and 5 cm spherical VOIs were outlined in the aortic arch and liver to measure the background uptake of physiologic blood volume and liver. We quantified SUVmax, MTV, and TLG for BMI, XL, and TD, and classified FDG PET/CT BMI status (BMhot) as binary (present/absent).

### 2.3. Bone Marrow Infiltration Assessment

Based on prior work [31], BMI was defined by focal or multifocal BM activity on FDG PET/CT with uptake greater than normal liver uptake and exceeding an SUV threshold of 4.0, unexplained by benign etiology on CT or clinical correlation. Confirmation required concordance with either iliac crest BMB findings or a treatment response on follow-up FDG PET/CT, indicated by decreased activity in suspected BM lesions. Negative BMI was defined by diffuse BM uptake or the absence of detectable uptake on baseline FDG PET/CT, the benign etiology of uptake, or persistent FDG uptake on evaluation PET/CT.

### 2.4. Statistical Analysis

The analysis was split to single time point and continuous survival analysis. For single time point, univariate logistic regression and associated statistical significance was used to identify variables contributing to OS3, OS5, and iCR. Correlation between variables was tested using Pearson’s R, and variables with univariate statistical strength below *p* < 0.1 and correlation below r < 0.6 (R^2^ < 0.36) were used in multivariate predictive models. Receiver–operator curves (ROC) were calculated for variables identified to bear statistical significance and a multinomial logistic regression model, and predictive power was compared using the area under the ROC curve (AUC). Specificity and sensitivity are reported at optimum operating points selected using Youden’s index, which was also used to determine thresholds for continuous variables in the odds ratio (OR) calculation and Kaplan–Meier analysis.

The log-rank difference of Kaplan–Meier survival curves was used as a measure of predictive variable statistical power in estimating survival. *p*-values based on maximum likelihood (ML) were used as a measure of statistical significance in a univariate Cox regression model, and the rate of change in model ML significance was used to evaluate the additional contribution of new variables in a multivariate Cox regression model. In univariate Cox models, hazard ratios (HRs) are reported for scaled continuous variables to account for their range. All statistical analysis was performed using R Statistical Software (v3.5.; R Core Team 2018). *p* < 0.05 was considered statistically significant.

## 3. Results

### 3.1. Patients’ Characteristics

**Figure 1:**
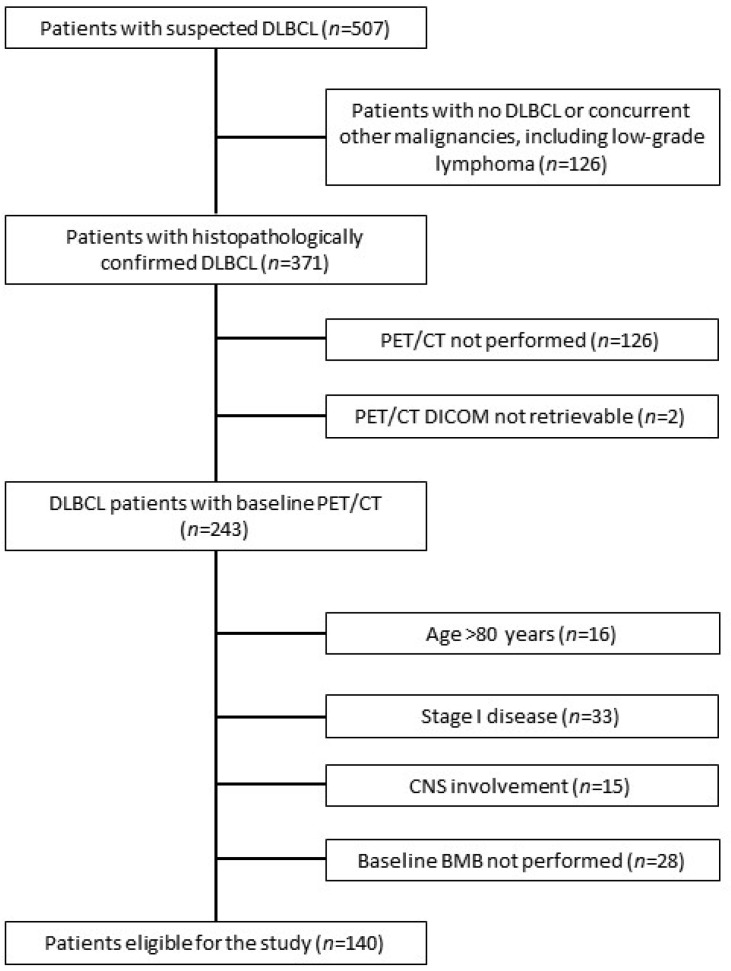
The flowchart of patients’ inclusion. DLBCL, diffuse large B-cell lymphoma; CNS, central nervous system; BMB, bone marrow biopsy.

A total of 140 patients were recruited for the study (Figure 1). Patients’ characteristics are presented in Table 1. The median SUVmax, MTV, and TLG in the XL of all patients were 26.24 (interquartile range (IQR): 19.81–33.46), 187.72 mL (IQR: 45.17–575.26 mL), and 1955.51 (IQR: 418.50–6687.60), respectively. BMhot was present in 35 (25%) patients with a median SUVmax, MTV, and TLG of 19.87 (IQR: 15.85–27.31), 33.12 mL (IQR: 12.71–110.86 mL), and 302.79 (IQR: 80.35–832.05), respectively. The median MTV and TLG of TD were 224.94 mL (IQR: 55.54–625.54 mL) and 2170.47 (IQR: 493.30–7401.10), respectively.

### 3.2. Survival Analysis

The median follow-up time was 47 months (range: 2–85 months; IQR: 29–63 months). Seventy-nine patients (56%) achieved iCR. Forty patients (29%) achieved partial response, followed by curative radiotherapy. Twenty-one patients (15%) progressed during initial treatment, with nine (6%) dying during treatment due to a progression of the disease. An additional 25 patients (18%), who had achieved iCR, died during the follow-up period (median time from the end of treatment to death: 17 months; IQR: 13–29 months).

In ROC analysis (Table 2), significant associations with iCR were observed for TD MTV (*p* = 0.001; AUC = 0.72), XL MTV (*p* = 0.001), BMhot (*p* = 0.008), IPI (*p* = 0.014), and disease stage (*p* = 0.028).

OS3 outcome was significantly associated with IPI (*p* = 0.000), WHO (*p* = 0.001), stage (*p* = 0.009), XL MTV (*p* = 0.02) and TD MTV (*p* = 0.023). Notably, IPI yielded the strongest association with OS3 (AUC = 0.75). Similarly, OS5 was significantly associated with IPI (*p* = 0.000; AUC = 0.78), WHO (*p* = 0.001), and stage (*p* = 0.018). Interestingly, PET parameters (XL MTV and TD MTV) were not significantly associated with OS5, although trends towards mild significance were observed. Additionally, tendencies toward mild significance were present for BMI SUVmax in both OS3 and OS5 outcomes (Table 3).

**Figure 2:**
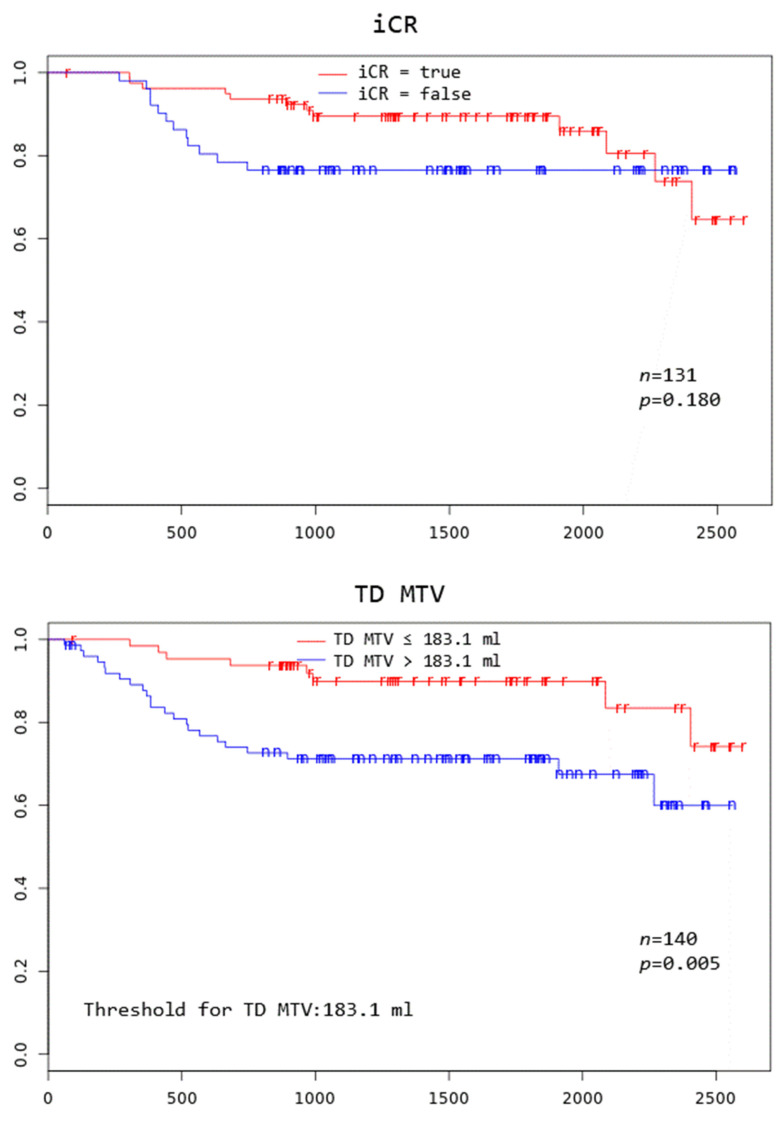

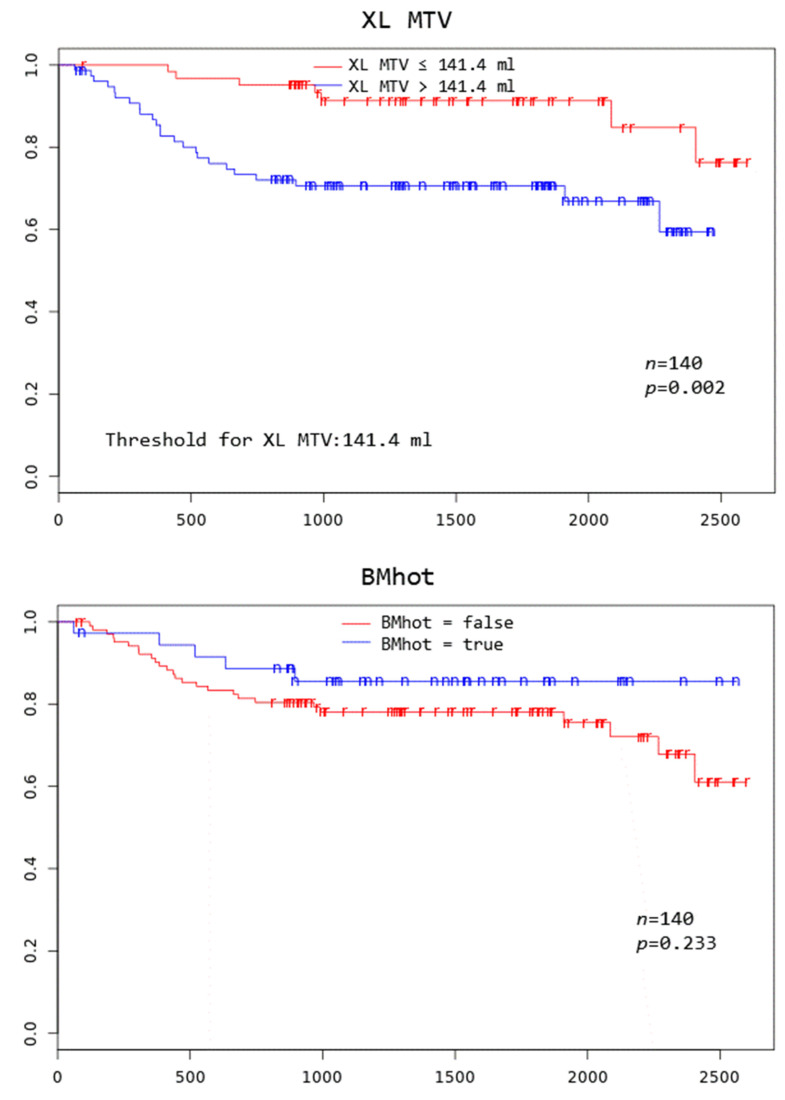

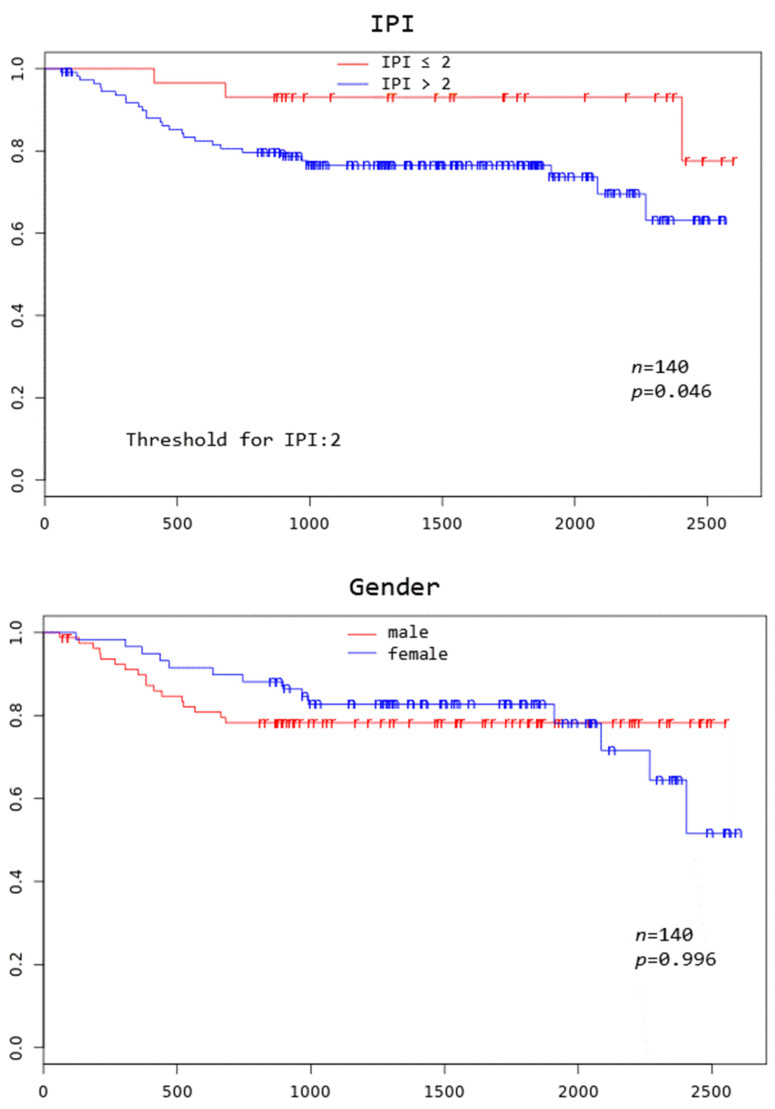

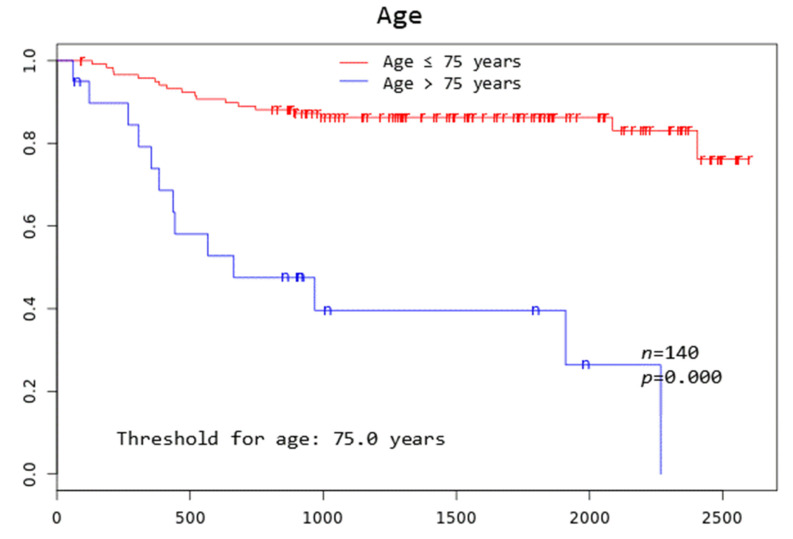
Kaplan–Meier curves to assess 3-year overall survival in DLBCL patients stratified by complete remission after first-line systemic treatment (iCR), total disease and lesions elsewhere metabolic tumor volume (TD MTV and XL MTV), bone marrow PET positivity (BMhot), International Prognostic Index score (IPI), gender, and age, respectively.

Patients with TD MTV exceeding 183.1 mL, XL MTV exceeding 141.1 mL, an IPI score greater than two, and age above 75 years all had a significantly worse overall survival at 3 years (*p* = 0.005, *p* = 0.002, *p* = 0.046, and *p* = 0.000, respectively) (Figure 2).

**Figure 3:**
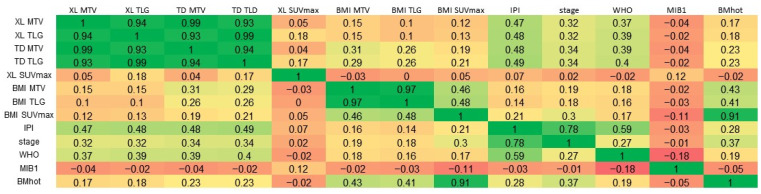
Correlations. A linear three-color gradient map is used to indicate a strong correlation (green), weak correlation (yellow) and a strong anti-correlation (red). MTV, metabolic tumor volume; TLG, total lesion glycolysis; TD, total disease; BMI, bone marrow involvement; XL, lesions elsewhere; IPI, International Prognostic Index score; WHO, World Health Organization performance status; MIB-1, MIB-1 immunohistochemical proliferation index; BMhot, bone marrow PET positivity.

A very strong positive correlation was observed between TD MTV and XL MTV (Pearson’s r = 0.99), and moderate positive correlations were found between the IPI and both TD MTV (r = 0.48) and XL MTV (r = 0.47) (Figure 3).

In univariate analysis for OS3, factors significantly predicting poorer survival were XL MTV (HR per 100 mL increment = 1.29; 95% confidence interval [CI]: 1.00–1.67; *p* = 0.049), BMI SUVmax (HR per one standard deviation (SD) increase = 0.56; 95% CI: 0.30–1.03; *p* = 0.02), higher IPI score (HR = 1.92; 95% CI: 1.42–2.60; *p* = 0.00), poorer WHO performance status (HR = 1.64; 95% CI: 1.19–2.26; *p* = 0.003), and advanced disease stage (HR = 2.40; 95% CI: 1.32–4.35; *p* = 0.004). By multivariate analysis, higher IPI (HR = 2.26; 95% CI: 1.48–3.45; *p* = 0.0001) and BMI SUVmax (HR = 0.91; 95% CI: 0.85–0.98; *p* = 0.008) were significant independent predictors for OS3; however, BMI SUVmax resulted in a negative coefficient and hence indicated a protective effect (Table 4).

**Figure 4:**
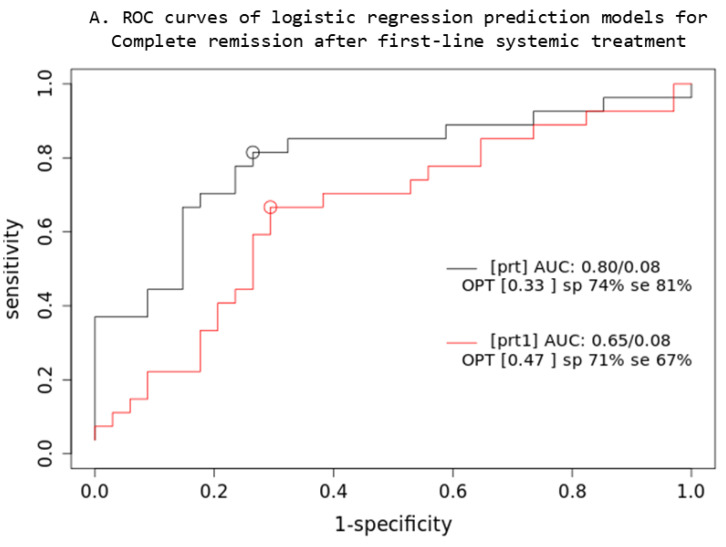

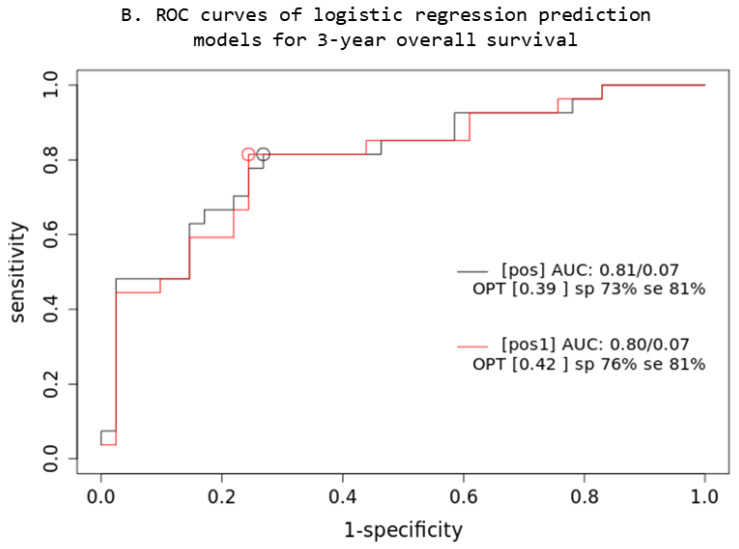
Prediction models: PET quantitative parameters ROC curve for complete remission after first-line systemic treatment (**A**) and 3-year overall survival (**B**). [prt] and [pos]: combined lesions elsewhere metabolic tumor volume + bone marrow involvement SUVmax + International Prognostic Index score prediction model; [prt1] and [pos1]: single-parameter International Prognostic Index score prediction model; AUC: area under the ROC curve; sp: specificity; se: sensitivity.

Based on the comparison of the AUC of two iCR logistic regression prediction models (Figure 4), a combined multivariable Cox model consisting of IPI + XL MTV + BMI SUVmax parameters (prt) significantly overperforms the single-parameter IPI (prt1) model (z = 5.22; *p* < 0.001; AIC: prt1 = 84, prt = 74), while no significant difference was observed in OS3 prediction.

## 4. Discussion

To the authors’ best knowledge, this is the first evaluation study conducting distinct volumetric analyses of BMI in DLBCL using manual volumetric FDG PET/CT analysis.

Our analyses revealed a paradox regarding BMI and its association with iCR and OS in DLBCL patients. Despite BMI, identified on FDG PET/CT, emerging as an important predictor of iCR, its significance waned when evaluated for OS3 and OS5 in ROC analysis, suggesting its limited utility as a diagnostic tool. Nonetheless, BMI demonstrated a significant positive association with OS3 in univariate Cox analysis but became inversely significant in the multivariate model after incorporating IPI.

One plausible explanation is that patients identified with BMI on FDG PET/CT and subsequently subjected to intensified therapeutic regimens may experience improved overall survival at later stages, attributed to intensified treatment. The discrepancy in the inverse significance of BMI in the multivariate model with included IPI suggests a potential confounding effect on the IPI score, which remained significant in all analyses (ROC, Kaplan–Meier, univariate, and multivariate Cox). Since IPI incorporates factors influencing treatment decisions, the increase in therapy intensity often results from a higher IPI [22]. Patients with higher IPI scores in our cohort, who were more likely to exhibit PET-positive BMI, have received a more intensive therapy, and while potentially improving overall survival, this IPI-guided intensified treatment could mask the direct negative impact of BMI itself, leading to the observed inverse association of BMI in the multivariate analysis.

This finding has not been previously reported, as no prior study has effectively examined the prognostic implications of volumetric BMI quantified through MTV and TLG. PET/CT tumor delineation is known to be time-intensive, particularly in patients with lymphoma, where multiple and heterogeneous nodal and extranodal masses are common [32]. Yamanaka et al. undertook a PET volumetric analysis of BMI in DLBCL but encountered technical challenges in distinguishing between reactive and pathological uptake, and abandoned the assessment of the BMI MTV prognostic value [19].

Other studies assessed the diagnostic value of BMI on FDG PET/CT using either a dichotomous categorization or absolute and relative values of uptake with SUVmax, a BM retention index, and BM-to-liver ratio [33,34,35]. A large study of 512 DLBCL patients reported FDG-positive BMI in relation to posterior iliac crest BMB as an independent predictor of a worse 2-year OS in both NCCN-IPI high, intermediate, and low subgroups [33]. Chang et al. reported that BM hypermetabolism over the sternum was an independent predictive factor for OS in DLBCL patients; however, PET-detected BMI was not significantly correlated with either IPI or histological BMI [36]. In contrast, a large multinational study by IAEA reported that only patients with BMI identified by both PET and BMB had significantly inferior survivals, with cases of BMI identified by just a single diagnostic modality having prognosis similar to patients with no evidence of BM disease irrespective of stage [37]. Several other studies also failed to confirm that FDG PET/CT BM status, unlike BMB, has prognostic implications [38,39,40]. These observations suggest that false-positive FDG PET/CT results occur in a considerable proportion of DLBCL patients because benign or other malignant BM lesions may also result in increased FDG uptake in BM [41]. To minimize these false-positive BM findings on PET to our analysis, we adopted stricter criteria for identifying BMI, requiring either concordant findings on BMB or a treatment response on evaluation PET scan.

Another reason for the mismatch of significance of BMI results between ROC, Kaplan–Meier, and COX analyses lies in the fact that Cox proportional hazards, by considering the entire survival curve, the continuous biomarker value, and the interplay with other variables, provide a more comprehensive assessment of the biomarker’s influence on survival outcomes compared to binary single-time-point Kaplan–Meier analysis [42].

Our investigation into tumor burden, as quantified by MTV on FDG PET/CT imaging, unveiled a significant but nuanced relationship. MTV exhibited statistical significance in ROC, Kaplan–Meier, and univariate Cox proportional hazard analysis. However, this significance waned in the multivariate Cox proportional hazard analysis, indicating that while MTV tumor burden alone is a noteworthy predictor, its impact may diminish when considered alongside other variables. Our data validate previous reports of IPI being a robust and reliable predictor of disease outcome, with its effect persisting even when FDG PET/CT variables are considered [43]. Similarly, the recently proposed International Metabolic Prognostic Index model for predicting outcomes in DLBCL patients, which incorporates total MTV with age and stage as continuous variables [44], proved not robust and did not outperform IPI in the real-world cohort [45]. On the other hand, several authors reported good predictive value of volumetric FDG PET/CT parameters: MTV was found to be an independent prognostic factor for the survival of DLBCL patients, and the combination of MTV and IPI improved OS3 prognostication [14], while a model incorporating IPI and baseline mediastinal-uptake corrected MTV provided an accurate prediction of the 3-year PFS [46]. Additionally, MTV, TLG, or SUVmax were reported to be independent predictors of survival in DLBCL patients [12,47,48]. Diverse results from these studies can be attributed to different segmentation thresholding methods, diverse inclusion criteria, and patients’ age and stage, and different treatment regimens included.

In the assessment of OS5 in DLBCL patients, our study found that, while the established clinical parameter IPI, along with some of its constituents (stage and WHO), emerged as the sole statistically significant prognostic factor, there was a notable trend towards significance for FDG PET/CT-defined tumor burden parameters. This suggests that while FDG PET/CT parameters did not reach statistical significance at the 5-year mark contrary to other reports [15], they may still hold potential prognostic value, albeit to a lesser degree. A possible conclusion might be that while FDG PET/CT parameters are useful for short- to mid-term prognostication, the IPI may be a more reliable indicator for the long-term timeframe in DLBCL patients.

Our study achieved an iCR rate of 56% (79 patients) after initial systemic treatment. This is slightly lower than some previously reported rates exceeding 75% [5]. This difference might be attributed to our stricter inclusion criteria, which excluded stage I patients, who typically respond well to treatment achieving CR. Notably, a similar CR rate of 52% was reported in a large study by Coiffier et al. with comparable inclusion criteria (stages II, III, and IV) [4]. As expected, our data show that lower tumor burden on FDG PET/CT scans (measured by TD MTV, XL MTV, and the absence of PET-detectable BMI), lower IPI, and earlier disease stage were all significantly associated with achieving an iCR.

This study has several limitations: the manual segmentation might have been biased by the involvement of a single reader, and data on subsequent therapies were not considered. Additionally, the single-center retrospective design of the study may impact generalizability. Future research should employ multiple blinded observers, utilize a prospective multi-center design, and consider data on subsequent therapies.

Overall, our study demonstrated that having a positive BMI in FDG PET/CT, although it initially indicates a worse outcome, allows appropriate treatment that may improve survival. Our findings contribute to the understanding of the independent effects of BMI and IPI-guided treatment on DLBCL patient outcomes. Further research may be needed to understand the specific time frames and clinical scenarios in which FDG PET/CT parameters provide the most valuable prognostic information for DLBCL patients. Moreover, exploring potential synergies between the IPI and FDG PET/CT parameters could enhance risk stratification and inform personalized treatment approaches in this patient population.

## 5. Conclusions

Baseline FDG PET/CT MTV is significantly associated with survival in DLBCL patients. BMI identified on FDG PET/CT allows appropriate treatment that may improve survival.

## Figures and Tables

**Table 1:** Clinical characteristics of patients.

Age (median) [years]	66 (20–80)
Gender, female/male, *n* (%)	59 (42%)/81 (58%)
IPI score, *n* (%)	
IPI Low risk group: 29 (21%)	
IPI Low-intermediate risk group: 30 (21%)	
IPI High-intermediate risk group: 33 (24%)	
IPI High risk group: 48 (34%)	
Stage at diagnosis, *n* (%)	II: 36 (26%)
	III: 20 (14%)
	IV: 84 (60%)
Chemotherapy regimen, *n* (%)	RCHOP: 108 (77%)
	REPOCH: 11 (8%)
	RCOEP: 6 (4%)
	Reduced RCHOP (mini-RCHOP 80%, 75%, 50%): 6 (4%)
	RACVBP: 6 (4%)
	Other: 3 (2%)
BMhot overall, *n* (%)	35 (25%)
Extranodal sites: 0, *n* (%)	24 (17%)
Extranodal sites: 1, *n* (%)	43 (31%)
Extranodal sites more than 1, *n* (%)	73 (52%)
Serum LDH elevated	76 (54%)
Proportion of patients receiving intensive immunochemotherapy regimens (RACVBP and REPOCH)	BMI present: 8/36 (22%) *p* = 0.041 BMI absent: 9/104 (9%)

IPI, International Prognostic Index; BMhot, bone marrow PET positivity; LDH, lactate dehydrogenase; BMI, bone marrow involvement.

**Table 2:** Receiver–operating characteristics analysis of prognostic factors for complete remission after initial systemic treatment.

Variable	(*n*)	AUC	*p*	Optimal Threshold	Sensitivity	Specificity
XL MTV	131	0.69	0.003 *	157.21 mL	61	71
TD MTV	131	0.71	0.001 *	157.21 mL	59	76
XL SUVmax	131	0.55	0.165	17.3	30	92
BMI MTV	131	0.61	0.154	>0	51	73
BMI SUVmax	131	0.6	0.017 *	>0	51	73
BMhot	131		0.014 *			
WHO	131	0.57	0.117	>1	68	51
IPI	131	0.63	0.014 *	>3	69	59
MIB-1	123	0.5	0.885	90	68	16
Stage	131	0.61	0.028 *	>4	79	49

* Factors were statistically significant. TD, total body; BMI, bone marrow involvement; XL, lesions elsewhere; BMhot, bone marrow PET positivity; WHO, World Health Organization performance status; IPI, International Prognostic Index; MIB-1, MIB-1 immunohistochemical proliferation index.

**Table 3:** Receiver–operating characteristics analysis of prognostic factors for 3- and 5-year overall survival.

Overall Survival at 3 Years							Overall Survival at 5 Years					
Variable	(*n*)	AUC	*p*	Optimal Threshold	Sensitivity	Specificity	(*n*)	AUC	*p*	Optimal Threshold	Sensitivity	Specificity
XL MTV	115	0.66	0.039 *	141.4 mL	51	82	72	0.66	0.122	273.84 mL	59	71
TD MTV	115	0.64	0.047 *	183.05 mL	49	79	72	0.66	0.117	273.84 mL	59	71
XL SUVmax	115	0.45	0.51	26.23	45	39	72	0.46	0.5	26.48	45	39
BMI MTV	115	0.46	0.816	>0	64	21	72	0.48	0.412	>0	64	21
BMISUVmax	115	0.44	0.138	>15.57	77	7	72	0.46	0.245	>0	64	21
BMhot	115		0.452				72		0.769			
WHO	115	0.7	0.001 *	>1	80	61	72	0.73	0.001 *	>1	82	61
IPI	115	0.75	<0.0001 *	>4	91	50	72	0.78	<0.0001 *	>2	57	89
MIB-1	107	0.49	0.619	90	81	30	68	0.49	0.474	90	80	33
stage	115	0.66	0.009 *	>4	53	86	72	0.65	0.018 *	>4	50	86

* Factors were statistically significant. TD, total body; BMI, bone marrow involvement; XL, lesions elsewhere; BMhot, bone marrow PET positivity; WHO, World Health Organization performance status; IPI, International Prognostic Index; MIB-1, MIB-1 immunohistochemical proliferation index.

**Table 4:** Univariate and multivariate Cox proportional hazard analysis for 3-year overall survival.

	Univariate				Multivariate	
Variable	Unit Change	HR (95%CI)	*p*	Coefficient	HR (95%CI)	*p*
XL MTV	812 mL ^¤^	1.29 (1.00–1.67)	0.049 *	###	###	0.51
XL MTV	100 mL	1.03 (1.00–1.07)	0.049 *	###	###	0.51
TD MTV	842 mL ^¤^	1.28 (0.97–1.68)	0.079			
XL SUVmax	11 ^¤^	0.93 (0.66–1.33)	0.71			
BMI MTV	142 mL ^¤^	0.44 (0.08–2.58)	0.37			
BMI SUVmax	10 ^¤^	0.56 (0.30–1.03)	0.022 *	−0.09	0.91 (0.85–0.98)	0.008 *
BMhot	1	0.44 (0.16–1.27)	0.10			
WHO	1	1.64 (1.19–2.26)	0.003 *	###	###	0.99
IPI	1	1.92 (1.42–2.60)	<0.0001 *	0.82	2.26 (1.48–3.45)	0.0001 *
MIB-1	13 ^¤^	0.83 (0.60–1.13)	0.23			
stage	1	2.40 (1.32–4.35)	0.004			

While XL MTV and WHO were included in the multivariate model, the resulting coefficients and associated HR were blinded by the ### character as they do not contribute to the multivariate model with a statistical significance. ^¤^ Unit change per one standard deviation increase. * Factors were statistically significant. ### excluded from analysis due to lack of statistical significance. MTV, metabolic tumor volume; TLG, total lesion glycolysis; TD, total disease; BMI, bone marrow involvement; XL, lesions elsewhere; BMhot, bone marrow PET positivity; WHO, World Health Organization performance status; IPI, International Prognostic Index score; MIB-1, MIB-1 immunohistochemical proliferation index.

## Data Availability

The raw data supporting the conclusions of this article will be made available by the authors on request.
